# Cyclo­hexa­none 2-nitro­phenyl­hydrazone

**DOI:** 10.1107/S1600536810016156

**Published:** 2010-05-08

**Authors:** Bao-he Yang, Jun-long Niu

**Affiliations:** a Department of Physics, Zhengzhou Normal University, Zhengzhou 450044, People’s Republic of China; b Department of Chemistry, Zhengzhou University, Zhengzhou 450052, People’s Republic of China

## Abstract

In the title Schiff base compound, C_12_H_15_N_3_O_2_, obtained from a condensation reaction of cyclo­hexa­none and 2-nitro­phenyl­hydrazine, the phenyl­hydrazone group is planar, the largest deviation from the mean plane being 0.0252 (12) Å, and the nitro fragment is twisted slightly with respect to the mean plane, making a dihedral angle of 6.96 (17)°. The cyclo­heaxanone ring displays a chair conformation. An intra­molecular N—H⋯O hydrogen bond helps to stabilize the mol­ecular structure.

## Related literature

For the important role played by hydrazone derivatives in the development of various proteins and enzymes, see: Kahwa *et al.* (1986 ▶); Santos *et al.* (2001 ▶). For puckering parameters, see Cremer & Pople (1975 ▶). For a related structure, see: Shan *et al.* (2003 ▶).
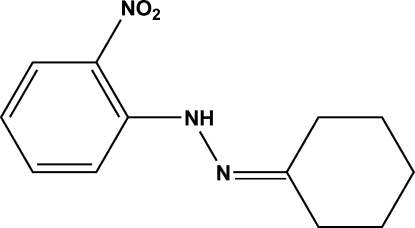


## Experimental

### 

#### Crystal data

C_12_H_15_N_3_O_2_
*M* *_r_* = 233.27
Monoclinic, 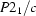

*a* = 8.519 (5) Å
*b* = 19.609 (7) Å
*c* = 7.822 (4) Å
β = 112.110 (7)°
*V* = 1210.6 (10) Å^3^
*Z* = 4
Mo *K*α radiation
μ = 0.09 mm^−1^
*T* = 293 K
0.23 × 0.20 × 0.19 mm

#### Data collection

Bruker SMART CCD area-detector diffractometer
Absorption correction: multi-scan (*SADABS*; Bruker, 1998 ▶) *T*_min_ = 0.973, *T*_max_ = 0.977
4958 measured reflections
2472 independent reflections
739 reflections with *I* > 2σ(*I*)
*R*_int_ = 0.035

#### Refinement

*R*[*F* ^2^ > 2σ(*F* ^2^)] = 0.035
*wR*(*F* ^2^) = 0.066
*S* = 0.64
2472 reflections
155 parameters
H-atom parameters constrained
Δρ_max_ = 0.09 e Å^−3^
Δρ_min_ = −0.11 e Å^−3^

### 

Data collection: *SMART* (Bruker, 1998 ▶); cell refinement: *SAINT* (Bruker, 1998 ▶); data reduction: *SAINT*; program(s) used to solve structure: *SHELXTL* (Sheldrick, 2008 ▶); program(s) used to refine structure: *SHELXL97* (Sheldrick, 2008 ▶); molecular graphics: *ORTEPIII* (Burnett & Johnson, 1996 ▶) and *ORTEP-3 for Windows* (Farrugia, 1997 ▶); software used to prepare material for publication: *SHELXTL*.

## Supplementary Material

Crystal structure: contains datablocks global, I. DOI: 10.1107/S1600536810016156/dn2561sup1.cif

Structure factors: contains datablocks I. DOI: 10.1107/S1600536810016156/dn2561Isup2.hkl

Additional supplementary materials:  crystallographic information; 3D view; checkCIF report

## Figures and Tables

**Table 1:** Hydrogen-bond geometry (Å, °)

*D*—H⋯*A*	*D*—H	H⋯*A*	*D*⋯*A*	*D*—H⋯*A*
N2—H2⋯O2	0.86	1.98	2.599 (2)	128

**Fig. 1.**
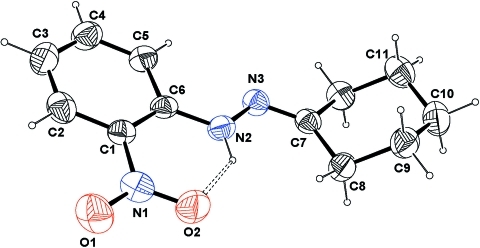
Molecular view of (I) with the atom labeling scheme. Displacement ellipsoids are drawn at the 30% probability level. H atoms are represented as small sphere of arbitrary radii. Intramolecular hydrogen bond is shown as dashed lines.

## supplementary crystallographic information

### Comment

The chemistry of Schiff base has attracted a great deal of interest in recent years. These compounds play an important role in the development of various proteins and enzymes (Kahwa *et al.*, 1986; Santos *et al.*, 2001). In this paper, we synthesized the title compound and reported its crystal structure.

In the title compound, the phenylhydrazone group is planar with the largest deviation from the mean plane being 0.0252 (12)Å, the nitro fragment is sligthly twisted with respect to this mean plane making a dihedral angle of 6.96 (17)° (Fig. 1). The cycloheaxanone displays a chair conformation as confirmed by the ring puckering parameters, θ= 5.6 (3)° and φ=195 (3)° (Cremer & Pople, 1975). The C-N and N-N distances within the hydrazone moity agree with related compound (Shan *et al.*, 2003).

Intramolecular N—H···O hydrogen bond stabilizes the crystal structure.

### Experimental

2-Nitrophenylhydrazine (1 mmol, 0.153 g) was dissolved in anhydrous ethanol (15 ml), The mixture was stirred for several minitutes at 351k, cyclohexanone (1 mmol, 0.098 g) in ethanol (8 mm l) was added dropwise and the mixture was stirred at refluxing temperature for 2 h. The product was isolated and recrystallized from methanol/dicholomethane(1:1), red single crystals of (I) was obtained after 3 d.

### Refinement

All H atoms were positioned geometrically and treated as riding on their parent atoms with C—H=0.93Å (aromatic), 0.97Å(methylene) and N—H=0.86 Å, with U_iso_(H)=1.2U_eq_(C,N).

### Crystal data

**Table:**

C_12_H_15_N_3_O_2_	*F*(000) = 496
*M**_r_* = 233.27	*D*_x_ = 1.280 Mg m^−^^3^
Monoclinic, *P*2_1_/*c*	Mo *K*α radiation, λ = 0.71073 Å
Hall symbol: -P 2ybc	Cell parameters from 666 reflections
*a* = 8.519 (5) Å	θ = 3.0–26.3°
*b* = 19.609 (7) Å	µ = 0.09 mm^−^^1^
*c* = 7.822 (4) Å	*T* = 293 K
β = 112.110 (7)°	Block, red
*V* = 1210.6 (10) Å^3^	0.23 × 0.20 × 0.19 mm
*Z* = 4	

### Data collection

**Table:**

Bruker SMART CCD area-detector diffractometer	2472 independent reflections
Radiation source: fine-focus sealed tube	739 reflections with *I* > 2σ(*I*)
graphite	*R*_int_ = 0.035
ω scans	θ_max_ = 26.4°, θ_min_ = 3.0°
Absorption correction: multi-scan (*SADABS*; Bruker, 1998)	*h* = −10→8
*T*_min_ = 0.973, *T*_max_ = 0.977	*k* = −24→23
4958 measured reflections	*l* = −8→9

### Refinement

**Table:**

Refinement on *F*^2^	Primary atom site location: structure-invariant direct methods
Least-squares matrix: full	Secondary atom site location: difference Fourier map
*R*[*F*^2^ > 2σ(*F*^2^)] = 0.035	Hydrogen site location: inferred from neighbouring sites
*wR*(*F*^2^) = 0.066	H-atom parameters constrained
*S* = 0.64	*w* = 1/[σ^2^(*F*_o_^2^) + (0.0244*P*)^2^] where *P* = (*F*_o_^2^ + 2*F*_c_^2^)/3
2472 reflections	(Δ/σ)_max_ = 0.001
155 parameters	Δρ_max_ = 0.09 e Å^−^^3^
0 restraints	Δρ_min_ = −0.11 e Å^−^^3^

### Special details

**Table:**

Geometry. All esds (except the esd in the dihedral angle between two l.s. planes) are estimated using the full covariance matrix. The cell esds are taken into account individually in the estimation of esds in distances, angles and torsion angles; correlations between esds in cell parameters are only used when they are defined by crystal symmetry. An approximate (isotropic) treatment of cell esds is used for estimating esds involving l.s. planes.
Refinement. Refinement of *F*^2^ against ALL reflections. The weighted *R*-factor wR and goodness of fit *S* are based on *F*^2^, conventional *R*-factors *R* are based on *F*, with *F* set to zero for negative *F*^2^. The threshold expression of *F*^2^ > σ(*F*^2^) is used only for calculating *R*-factors(gt) etc. and is not relevant to the choice of reflections for refinement. *R*-factors based on *F*^2^ are statistically about twice as large as those based on *F*, and *R*- factors based on ALL data will be even larger.

### Fractional atomic coordinates and isotropic or equivalent isotropic displacement parameters (Å^2^)

**Table:**

	*x*	*y*	*z*	*U*_iso_*/*U*_eq_	
O1	−0.0513 (2)	0.70011 (9)	0.6282 (2)	0.1024 (6)	
O2	0.0322 (2)	0.60900 (8)	0.7886 (2)	0.0979 (6)	
N1	0.0275 (3)	0.64621 (11)	0.6599 (3)	0.0736 (6)	
N2	0.19470 (18)	0.51469 (9)	0.6890 (2)	0.0621 (5)	
H2	0.1484	0.5239	0.7669	0.075*	
N3	0.2696 (2)	0.45142 (10)	0.6925 (2)	0.0614 (5)	
C1	0.1174 (3)	0.62570 (13)	0.5443 (3)	0.0567 (6)	
C2	0.1241 (3)	0.67296 (11)	0.4156 (3)	0.0716 (6)	
H2B	0.0729	0.7153	0.4079	0.086*	
C3	0.2056 (3)	0.65756 (14)	0.2998 (3)	0.0802 (7)	
H3B	0.2101	0.6890	0.2128	0.096*	
C4	0.2813 (3)	0.59436 (15)	0.3146 (3)	0.0795 (7)	
H4A	0.3373	0.5836	0.2365	0.095*	
C5	0.2757 (2)	0.54760 (11)	0.4404 (3)	0.0664 (6)	
H5A	0.3268	0.5053	0.4454	0.080*	
C6	0.1950 (2)	0.56162 (12)	0.5625 (3)	0.0540 (5)	
C7	0.2737 (2)	0.41110 (11)	0.8199 (3)	0.0577 (6)	
C8	0.2140 (3)	0.42256 (10)	0.9746 (3)	0.0716 (6)	
H8A	0.1145	0.3949	0.9554	0.086*	
H8B	0.1826	0.4700	0.9762	0.086*	
C9	0.3519 (3)	0.40421 (11)	1.1584 (3)	0.0783 (7)	
H9A	0.4436	0.4370	1.1866	0.094*	
H9B	0.3064	0.4071	1.2547	0.094*	
C10	0.4207 (3)	0.33364 (11)	1.1574 (3)	0.0890 (7)	
H10A	0.3317	0.3004	1.1402	0.107*	
H10B	0.5111	0.3247	1.2755	0.107*	
C11	0.4880 (3)	0.32612 (11)	1.0044 (3)	0.0859 (7)	
H11A	0.5276	0.2798	1.0032	0.103*	
H11B	0.5832	0.3567	1.0270	0.103*	
C12	0.3504 (3)	0.34250 (10)	0.8190 (3)	0.0722 (6)	
H12A	0.3981	0.3415	0.7243	0.087*	
H12B	0.2626	0.3080	0.7891	0.087*	

### Atomic displacement parameters (Å^2^)

**Table:**

	*U*^11^	*U*^22^	*U*^33^	*U*^12^	*U*^13^	*U*^23^
O1	0.1178 (15)	0.0768 (11)	0.1168 (14)	0.0302 (11)	0.0489 (12)	−0.0068 (10)
O2	0.1121 (15)	0.1044 (14)	0.1003 (13)	0.0261 (10)	0.0662 (12)	0.0126 (11)
N1	0.0680 (15)	0.0692 (16)	0.0808 (15)	0.0008 (12)	0.0246 (14)	−0.0132 (13)
N2	0.0626 (14)	0.0648 (12)	0.0657 (12)	0.0003 (10)	0.0319 (11)	0.0026 (10)
N3	0.0617 (12)	0.0553 (12)	0.0665 (12)	0.0020 (10)	0.0234 (10)	−0.0003 (10)
C1	0.0491 (16)	0.0625 (16)	0.0589 (14)	−0.0032 (13)	0.0207 (13)	−0.0044 (14)
C2	0.0627 (17)	0.0662 (16)	0.0706 (16)	−0.0056 (13)	0.0077 (14)	−0.0003 (15)
C3	0.0862 (19)	0.081 (2)	0.0700 (17)	−0.0111 (16)	0.0258 (15)	0.0096 (15)
C4	0.0774 (19)	0.096 (2)	0.0720 (17)	−0.0038 (16)	0.0365 (15)	0.0006 (16)
C5	0.0650 (17)	0.0712 (17)	0.0686 (15)	0.0002 (12)	0.0314 (14)	−0.0004 (14)
C6	0.0413 (14)	0.0648 (17)	0.0554 (14)	−0.0098 (13)	0.0175 (12)	−0.0061 (13)
C7	0.0491 (14)	0.0576 (15)	0.0613 (14)	−0.0049 (12)	0.0150 (12)	−0.0030 (13)
C8	0.0701 (17)	0.0780 (16)	0.0674 (15)	−0.0056 (12)	0.0266 (15)	0.0073 (13)
C9	0.0760 (18)	0.0897 (17)	0.0644 (16)	−0.0126 (14)	0.0210 (15)	0.0007 (14)
C10	0.0890 (19)	0.0827 (18)	0.0785 (17)	−0.0061 (15)	0.0123 (15)	0.0160 (15)
C11	0.0792 (19)	0.0711 (16)	0.0945 (19)	0.0097 (14)	0.0181 (18)	0.0029 (15)
C12	0.0732 (17)	0.0607 (15)	0.0778 (16)	−0.0054 (13)	0.0226 (15)	−0.0030 (13)

### Geometric parameters (Å, °)

**Table:**

O1—N1	1.2262 (19)	C7—C8	1.496 (2)
O2—N1	1.2319 (19)	C7—C12	1.497 (2)
N1—C1	1.445 (2)	C8—C9	1.518 (3)
N2—C6	1.352 (2)	C8—H8A	0.9700
N2—N3	1.3906 (18)	C8—H8B	0.9700
N2—H2	0.8600	C9—C10	1.504 (2)
N3—C7	1.262 (2)	C9—H9A	0.9700
C1—C2	1.385 (2)	C9—H9B	0.9700
C1—C6	1.402 (2)	C10—C11	1.516 (3)
C2—C3	1.366 (3)	C10—H10A	0.9700
C2—H2B	0.9300	C10—H10B	0.9700
C3—C4	1.381 (3)	C11—C12	1.517 (3)
C3—H3B	0.9300	C11—H11A	0.9700
C4—C5	1.359 (2)	C11—H11B	0.9700
C4—H4A	0.9300	C12—H12A	0.9700
C5—C6	1.398 (2)	C12—H12B	0.9700
C5—H5A	0.9300		
			
O1—N1—O2	121.5 (2)	C7—C8—H8A	109.5
O1—N1—C1	119.5 (2)	C9—C8—H8A	109.5
O2—N1—C1	119.0 (2)	C7—C8—H8B	109.5
C6—N2—N3	119.62 (17)	C9—C8—H8B	109.5
C6—N2—H2	120.2	H8A—C8—H8B	108.1
N3—N2—H2	120.2	C10—C9—C8	112.17 (17)
C7—N3—N2	116.77 (17)	C10—C9—H9A	109.2
C2—C1—C6	121.8 (2)	C8—C9—H9A	109.2
C2—C1—N1	116.4 (2)	C10—C9—H9B	109.2
C6—C1—N1	121.8 (2)	C8—C9—H9B	109.2
C3—C2—C1	120.2 (2)	H9A—C9—H9B	107.9
C3—C2—H2B	119.9	C9—C10—C11	110.93 (18)
C1—C2—H2B	119.9	C9—C10—H10A	109.5
C2—C3—C4	118.7 (2)	C11—C10—H10A	109.5
C2—C3—H3B	120.6	C9—C10—H10B	109.5
C4—C3—H3B	120.6	C11—C10—H10B	109.5
C5—C4—C3	121.6 (2)	H10A—C10—H10B	108.0
C5—C4—H4A	119.2	C10—C11—C12	110.38 (18)
C3—C4—H4A	119.2	C10—C11—H11A	109.6
C4—C5—C6	121.5 (2)	C12—C11—H11A	109.6
C4—C5—H5A	119.3	C10—C11—H11B	109.6
C6—C5—H5A	119.3	C12—C11—H11B	109.6
N2—C6—C5	120.2 (2)	H11A—C11—H11B	108.1
N2—C6—C1	123.6 (2)	C7—C12—C11	111.55 (17)
C5—C6—C1	116.2 (2)	C7—C12—H12A	109.3
N3—C7—C8	128.90 (19)	C11—C12—H12A	109.3
N3—C7—C12	116.2 (2)	C7—C12—H12B	109.3
C8—C7—C12	114.9 (2)	C11—C12—H12B	109.3
C7—C8—C9	110.68 (17)	H12A—C12—H12B	108.0

### Hydrogen-bond geometry (Å, °)

**Table:**

*D*—H···*A*	*D*—H	H···*A*	*D*···*A*	*D*—H···*A*
N2—H2···O2	0.86	1.98	2.599 (2)	128

## References

[bb1] Bruker (1998). *SMART*, *SAINT* and *SADABS* Bruker AXS Inc., Madison, Wisconsin, USA.

[bb2] Burnett, M. N. & Johnson, C. K. (1996). *ORTEPIII* Report ORNL-6895. Oak Ridge National Laboratory, Tennessee, USA.

[bb3] Cremer, D. & Pople, J. A. (1975). *J. Am. Chem. Soc.* **97**, 1354–1358.

[bb4] Farrugia, L. J. (1997). *J. Appl. Cryst.* **30**, 565.

[bb5] Kahwa, I. A., Selbin, I., Hsieh, T. C. Y. & Laine, R. A. (1986). *Inorg. Chim. Acta*, **118**, 179–185.

[bb6] Santos, M. L. P., Bagatin, I. A., Pereira, E. M. & Ferreira, A. M. D. C. (2001). *J. Chem. Soc. Dalton Trans.* pp. 838–844.

[bb7] Shan, S., Xu, D.-J. & Hu, W.-X. (2003). *Acta Cryst.* E**59**, o1173–o1174.

[bb8] Sheldrick, G. M. (2008). *Acta Cryst.* A**64**, 112–122. 10.1107/S0108767307043930 18156677

